# Supplementary material for: Plasma membrane damage causes NLRP3 activation and pyroptosis during Mycobacterium tuberculosis infection
Source: Nat Commun. 2020 May 8;11:2270. doi: 10.1038/s41467-020-16143-6 (PMC7210277; doi:10.1038/s41467-020-16143-6)
Supplement: Supplementary file 1 — Supplementary figures and table [file 41467_2020_16143_MOESM1_ESM.pdf]

Supplementary Information

Plasma membrane damage causes NLRP3 activation and pyroptosis during *Mycobacterium tuberculosis* infection

Beckwith, Beckwith et al.

This PDF file includes:

Supplementary Figures 1-7

Supplementary Table 1

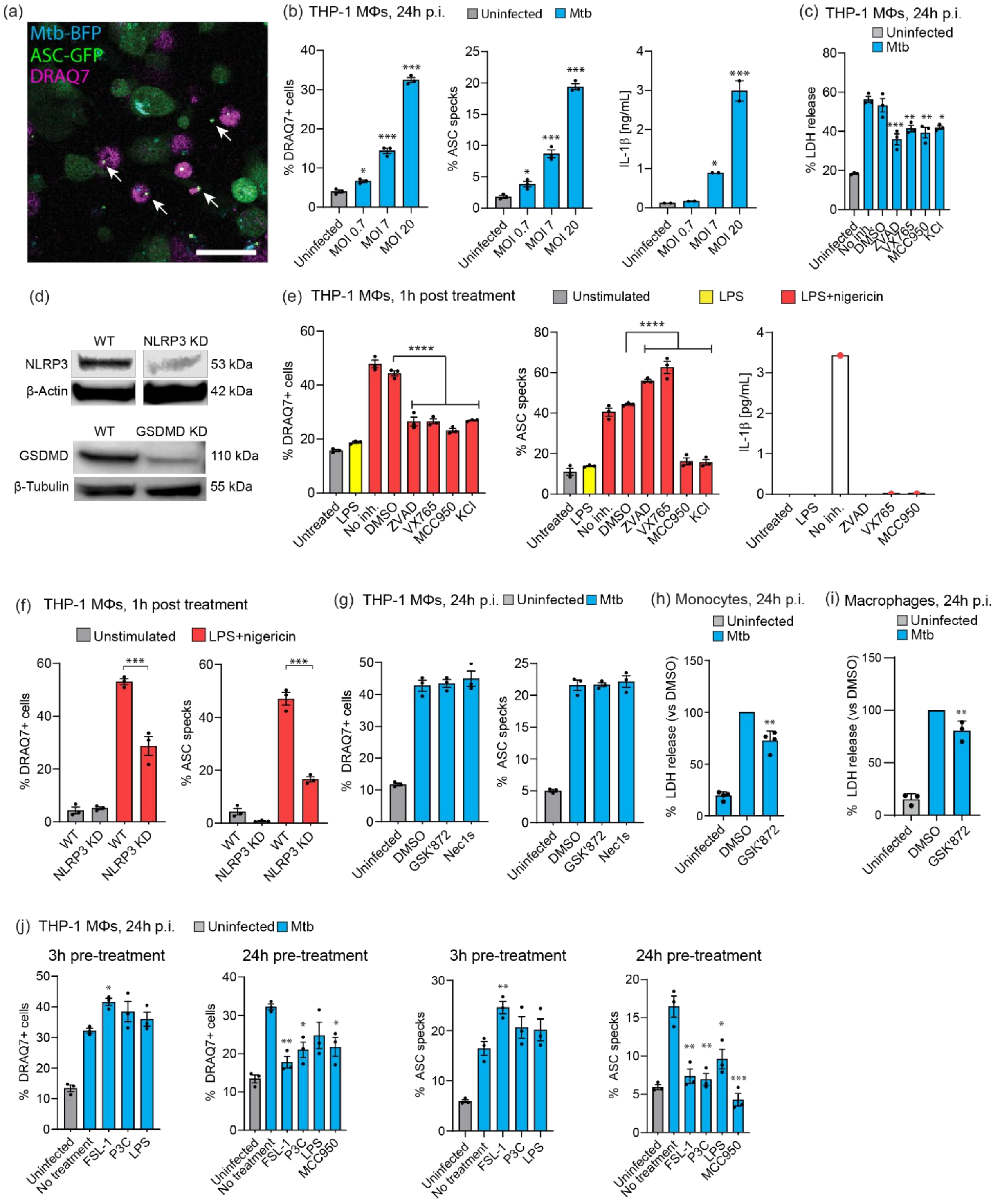

Supplementary Figure 1, related to main Figure 1. (**a**) Mtb (cyan) infection induced the assembly of ASC specks, observed as bright green spots (white arrows). Diffuse cytosolic green indicates live cells while DRAQ7 labels dead cells (magenta). Scale bar 10 μm. Data representative of 30 independent experiments. (**b**) THP1 ASC-GFP cells were infected with the indicated MOI of Mtb-BFP. DRAQ7+ cells and ASC specks were quantified, and IL-1β release determined by ELISA at 24h p.i. (**c**) Cell death of THP1 cells infected with Mtb-BFP and treated with inhibitors as indicated, measured by LDH cytotoxicity assay at 24h p.i. (**d**) THP1 ASC-mNeonGreen (WT) cells and WT cells depleted of NLRP3 or

GSDMD were lysed and protein levels measured by western blotting. Data representative of 2 independent experiments. (**e**) THP1 ASC-GFP cells were stimulated with LPS and nigericin, in the presence of inhibitors as indicated. DRAQ-7+ cells and ASC-specks were quantified at 24h p.i.. IL-1β in the supernatant was quantified by ELISA. (**f**) THP1 ASC-mNeonGreen cells (WT) and NLRP3 KD cells were stimulated by LPS and nigericin, and DRAQ7+ cells and ASC specks were quantified 1h post stimulation. (**g**) THP1 ASC-GFP cells were treated by inhibitors as indicated and infected by Mtb-BFP. DRAQ7+ cells and ASC specks were quantified 24h p.i.. (**h**) Primary human monocytes were treated with GSK'872 and infected with Mtb, and LDH release was determined 24h p.i.. (**i**) Primary human macrophages were treated with GSK'872 and infected with Mtb cultured without tween-80. LDH release was determined 24h p.i.. (**j**) THP1 ASC-GFP cells were stimulated for 3h or 24h prior to infection with Mtb-BFP, and DRAQ7+ cells and ASC specks were quantified 24h p.i.. In (**b**), (**c**), (**e**)-(**g**), (**h**)-(**i**), n>2000 cells per condition in triplicate, bars indicate mean±s.e.m., and data is representative of 2 independent experiments. Data in (**h**) from 4 donors and data in (**i**) from 3 donors. *p<0.05, **p<0.01, ***p<0.001, ****p<0.0001 by one-way ANOVA with two-sided Dunnett's test for (**b**)-(**c**), (**e**)-(**g**) and (**j**) and **p<0.01 by repeated measures ANOVA with two-sided Dunnett's test in (**h**) and (**i**), bars indicate mean±s.d. in both. Source data are provided as a Source Data file.

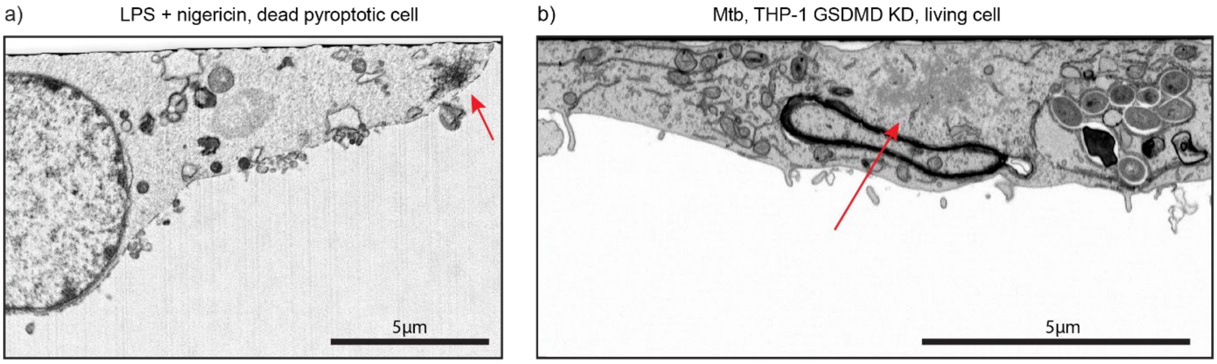

Supplementary Figure 2, related to main Figure 2. Single slices from FIB-SEM tomography of (**a**) THP1 ASC-GFP cell treated with LPS and nigericin. Data representative of 2 independent experiments. (**b**) THP1 ASC-mNeonGreen knocked down in GSDMD, infected with Mtb-BFP and fixed after ASC speck formation while the cell was still alive (DRAQ7 negative and normal morphology). ASC specks indicated by arrows. Data representative of 2 independent experiments.

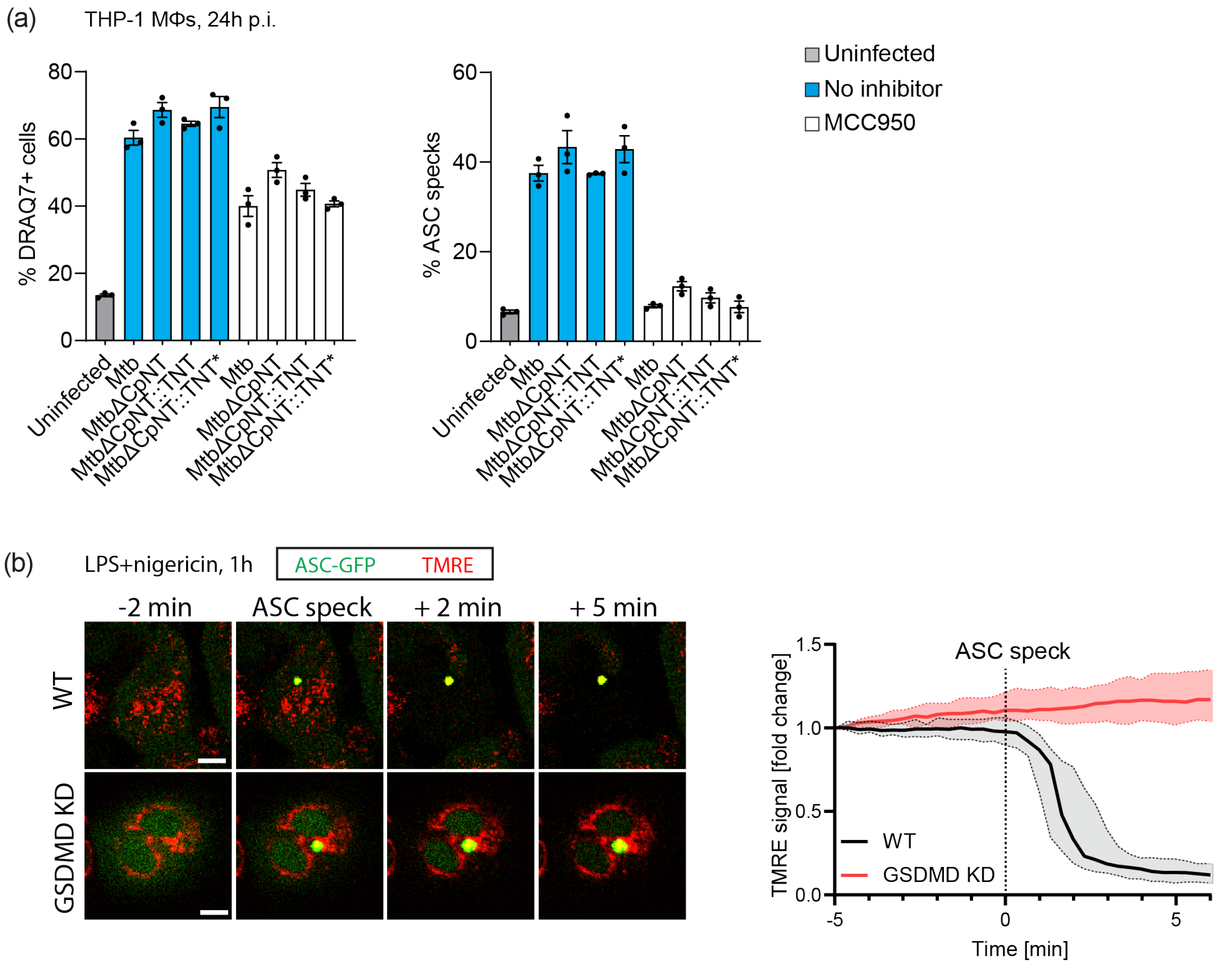

Supplementary Figure 3, related to main Figure 3. (**a**) THP1 ASC-GFP cells were infected with Mtb, MtbΔ*cpnT*, MtbΔ*cpnT*::*cpnT* and MtbΔ*cpnT*::*cpnT** (catalytically inactive TNT) in the absence or presence of MCC950. DRAQ7+ cells and ASC specks were quantified 24h p.i. for n>2000 cells per condition in triplicate. Mean±s.e.m. shown. (**b**) THP1 ASC-GFP cells (WT) or WT cells depleted for GSDMD were labelled with TMRE, treated with LPS and nigericin and imaged by time-lapse microscopy. Representative images of TMRE (red) and ASC (green) during ASC speck formation are shown. Quantification of TMRE intensity in single cells during ASC speck formation in WT (n=67 cells) and GSDMD KD cells (n=44 cells). Median±IQR shown. Scale bars 10 µm. Source data are provided as a Source Data file.

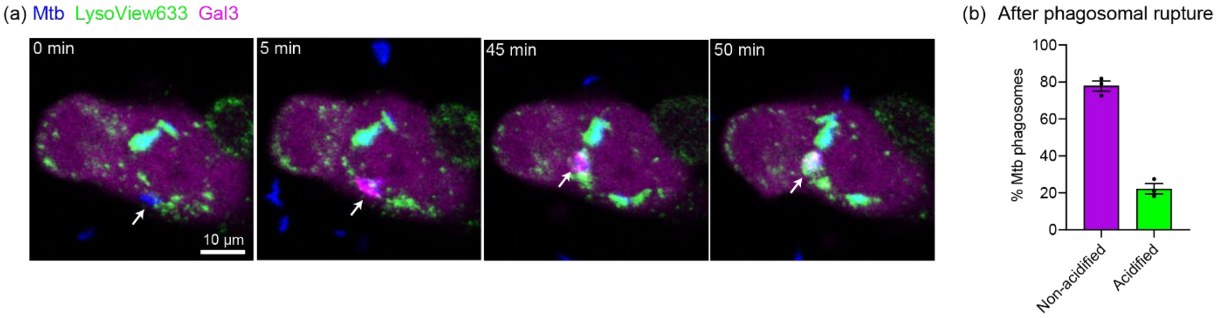

Supplementary Figure 4, related to main Figure 4. (**a**) Images from confocal time-lapse microscopy of THP1-Gal3-mScarlet cells labelled with LysoView633 and infected by Mtb-BFP. Arrows indicate Mtb (blue) -containing phagosomes recruiting Gal-3 (magenta) and LysoView633 (green). Data representative of n>100 events in 3 independent experiments. (**b**) Proportion of Mtb phagosomes gaining a LysoView signal indicating acidification after a Gal-3 event, within 24h or until cell death occurred. n=168 events from 3 independent experiments. Source data are provided as a Source Data file.

(a)

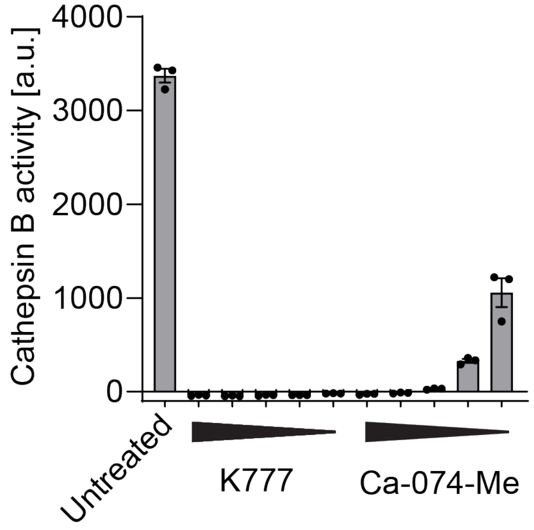

(b)

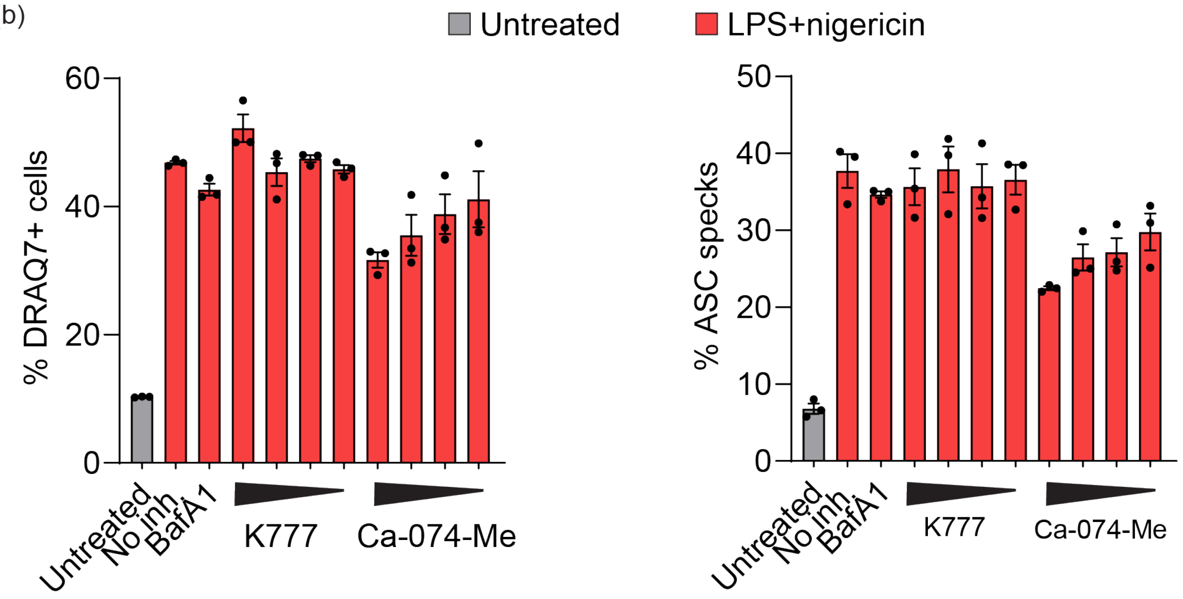

Supplementary Figure 5, related to main Figure 5. (**a**) Cathepsin B activity measured by the rate (V_{max}) of conversion of the cathepsin B substrate Z-RR-AMC in total cell lysates without or with 1h pre-treatment by cathepsin inhibitors K777 (30, 15, 7.5, 3.75, 1.6 µM) or Ca-074-Me (30, 15, 7.5, 3.75, 1.6 µM) for 1h. Mean±s.e.m. of technical triplicates shown. (**b**) Dose response of LPS and nigericin-treated THP-1 macrophages to BafA1 (50 nM), K777 (30, 15, 7.5, 3.75 µM) and Ca-074-Me (30, 15, 7.5, 3.75 µM). Cells were primed with 10ng LPS for 3h, pre-treated with inhibitors for 30min than treated with 5µM nigericin for 2h. Mean±s.e.m. shown. n>2000 cells per condition in triplicate, representative of 2 independent experiments. Source data are provided as a Source Data file.

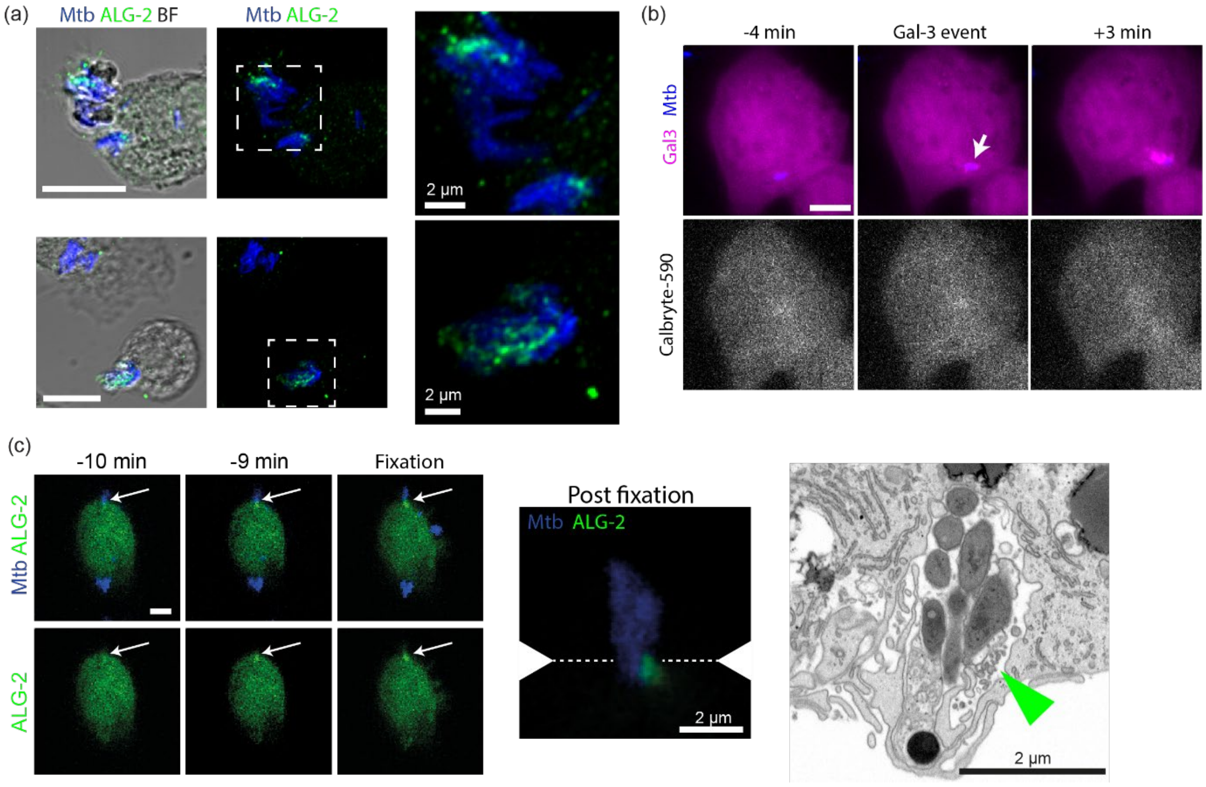

Supplementary Figure 6, related to main Figure 6. (**a**) Images of Mtb-localized ALG-2 (green) events labelled by immunofluorescence in fixed primary human macrophages infected with Mtb auxotroph (blue). Data representative of 2 independent experiments. (**b**) Representative images from time-lapse microscopy of Ca^{2+} sensitive Calbryte-590 (grey) signal during an Mtb (blue) localized Gal-3 (magenta) event in THP1 Gal-3-SNAP (SiR) cells. Arrows indicate Gal-3 event. Data representative of n>10 cells in 5 independent experiments. (**c**) Time-lapse confocal imaging of Mtb-localized ALG-2 event indicated by arrow in THP-1 mNeonGreen-ALG-2 cells infected by Mtb-BFP. After time-lapse imaging, cells were fixed and imaged by Airyscan and FIB-SEM tomography. The FIB-SEM imaging plane is perpendicular to the confocal image, indicated by triangles and dashed lines. Location of ALG-2 on confocal image is indicated by green triangle in SEM micrograph. All scale bars 10 µm unless otherwise specified. Data representative of 2 independent experiments.

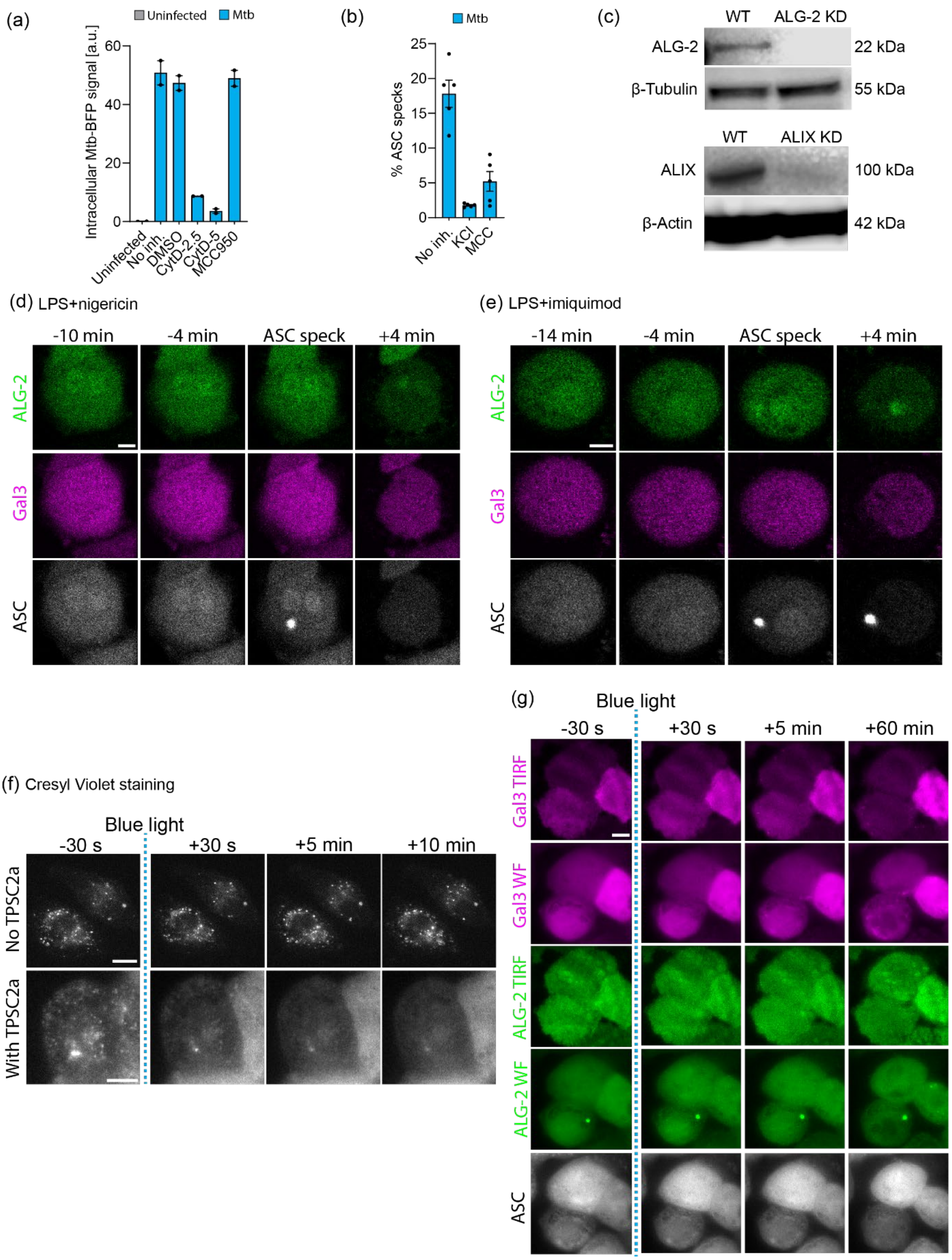

Supplementary Figure 7, related to main Figure 7. (**a**) The effect of the phagocytosis inhibitor cytochalasin D (cytoD) at the indicated concentrations (in µM) on inhibiting the uptake of Mtb by THP1 cells, assessed by measuring Mtb-BFP fluorescence inside an intracellular region defined by the cytosolic ASC-GFP signal in living THP1 cells. n>2000 cell per condition in triplicate. Mean±s.e.m. shown. (**b**) Quantification of ASC speck formation from 24h time-lapse experiments of THP1

mNeonGreen-ALG-2/Gal3-mScarlet/ASC-mIRFP670 treated as indicated and infected by Mtb-BFP, corresponding to experiment in Figure 7. n>200 cells analysed per condition, mean±s.d. of 5 fields of view shown. (**c**) ALG-2 or ALIX protein levels in lysates of THP1 ASC-mNeonGreen (WT) or ASC-mNeonGreen cells depleted of ALG-2 or ALIX by CRISPR-Cas9. (**d**)-(**e**) Representative time-lapse images of THP1 ALG2-mNeonGreen (green)/Gal3-mScarlet (magenta)/ASC-mIRFP670 (grey) cells pre-treated with LPS and imaged during treatment with nigericin (**d**) or imiquimod (**e**). Representative of n=100 (d) and n=132 (e) similar events. (**f**) THP1 cells with lysosomes labelled with Cresyl Violet (grey) and pulsed (16h) and chased (4h) with the photosensitizer $TPCS_{2a}$, then exposed to a 405 nm laser pulse in a widefield microscope to cause lysosomal damage and imaged by time-lapse microscopy. Representative of n>50 cells in 5 independent experiments. (**g**) THP1 ALG-2/Gal-3/ASC cells pulsed and chased with $TPCS_{2a}$, exposed to 405 nm blue light, and imaged for 60 minutes by simultaneous TIRF and widefield time-lapse microscopy. Representative of n>30 cells in 5 independent experiments. All scale bars 10 µm unless otherwise specified. Source data are provided as a Source Data file.

Supplementary Table 1

REAGENTS TABLE

REAGENT or RESOURCE	SOURCE	IDENTIFIER
**Antibodies**		
Rabbit mAb to human NLRP3	CST	Cat #13158S;<br>RRID:AB_2798134
Rabbit pAb to human GSDMD	Novus Bio	NBP2-33422;<br>RRID:AB_2687913
Rabbit pAb to human ALG-2	ThermoFisher	PA5-27639:<br>RRID:AB_2545115
Rabbit pAb to human ALIX	Novus Bio	NBP1-90201;<br>RRID:AB_11023702
ALG-2 Antibody (H-11)	Santa Cruz	Cat #sc-376950
Rabbit anti-Mouse IgG (H+L), Superclonal™ Recombinant Secondary Antibody, Alexa Fluor 647	ThermoFisher	Cat #A27029
**Bacterial and Virus Strains**		
Mtb H37Rv	ATCC	Cat #27294
Mtb H37RvΔRD1	Prof. R. Brosch	
Mtb H37Rv mc^{2}6206	Prof. W. Jacobs	(Jain *et al.*, 2014)
Mtb H37Rv::EBFP2	This work	
Mtb H37RvΔRD1::EBFP2	This work	
Mtb H37Rv mc^{2}6206::EBFP2	This work	
Mtb H37RvΔCpNT	Prof. M. Niederweis	(Danilchanka *et al.*, 2014)
Mtb H37RvΔCpNT::TNT	Prof. M. Niederweis	(Danilchanka *et al.*, 2014)
MtbH37Rv ΔCpNT::TNT* (catalytically inactive)	Prof. M. Niederweis	(Danilchanka *et al.*, 2014)
**Chemicals, Peptides, and Recombinant Proteins**		
Phorbol 12-myristate 13-acetate (PMA)	Sigma-Aldrich	Cat # P1585
Lymphoprep		
zVAD-FMK	Invivogen	Cat # tlrl-vad
VX-765	Invivogen	Cat # inh-vx765i
MCC950	Sigma-Aldrich	Cat #PZ0280
Potassium chloride	Sigma-Aldrich	Cat #P4504
Nec-1s	BioVision	Cat # 2263
GSK'872	BioVision	Cat # 2673
Cyclosporin A	Sigma-Aldrich	Cat # 30024
K777	Adipogen	Cat #AG-CR1-0158-M001
Ca-074-Me	Merck	Cat # 205531
Bafilomycin A1	Sigma-Aldrich	Cat # B1793
Cytochalasin D	Sigma-Aldrich	Cat #C8273
DRAQ7	Biolegend	Cat #424001
Tetramethylrhodamine ethyl ester perchlorate (TMRE)	Sigma-Aldrich	Cat # 87917
LysoView 633	Biotium	Cat # 70058
Calbryte-590	AAT Bioquest	Cat # 20700
SNAP-Cell 647-SiR	New England Biolabs	Cat # S9102S
Cresyl Violet	Sigma-Aldrich	Cat # C5042
$TPSC_{2a}$	PCI Biotech	
Z-Arg-Arg-7-amido-4-methylcoumarin hydrochloride	Sigma-Aldrich	Cat # C5429
FAM-YVAD-FMK	ImmunoChemistry Technologies	Cat #97
Recombinant Human GM-CSF	Peprotech	Cat #300-03
GM-CSF	Gibco-Thermo Fisher	Cat #PHC2011
**Critical Commercial Assays**		

Human IL-1β DuoSet ELISA	R&D systems	Cat # DY201
LDH Cytotoxicity Detection Kit	Takara Bio	Cat # MK401
CD14 MicroBeads UltraPure, human	Miltenyi Biotech	Cat #130-118-906
**Experimental Models: Cell Lines**		
THP-1	ATCC	Cat#TIB-202 RRID:CVCL_0006
THP-1 ASC-GFP	Prof. Emad Alnemri	(Fernandes-Alnemri *et al.*, 2007)
HEK293T	Thermo Scientific Open Biosystems	Cat#HCL4517
Human PBMC	St. Olavs blood bank, in-house recruited volunteers.	
**Oligonucleotides (all from ThermoFisher)**		
GGACAGGCAAAGATCGCAGG		GSDMD sgRNA
GCTGCAAGCTGGCCAGGTAC		NLRP3 sgRNA
TGCCGGACCAGAGCTTCCTG		ALG-2 sgRNA
CTTAAGTCGAGAGCCGACCG		ALIX sgRNA
GGGGACAAGTTTGTACAAAAAAGCAGGCTTC		attB1_fw
GGGGACAACTTTGTATACAAAAGTTGTC		attB5_fw
GGGGACCACTTTGTACAAGAAAGCTGGGTT		attB2_rv
GGGGACAACTTTTGTATACAAAGTTGC		attB5r_rv
AAAGCAGGCTTCATGGTGAGCAAGGGCGAG		attB1_EGFP_fw
TGTATACAAAGTTGCCTTGTACAGCTCGTCCATGCC		attB5r_EGFP_rv
GTATACAAAAGTTGTCGTGAGCAAGGGCGAGGAG		attB5_EGFP_fw
GAAAGCTGGGTTTTACTTGTACAGCTCGTCCATGCC		attB2_EGFP_rv
AAAGCAGGCTTCATGGACAAAGACTGCGAAATGAAGC		attB1_SNAPf
TGTATACAAAGTTGCACCCAGCCCAGGCTTG		attB5r_SNAPf
GTATACAAAAGTTGTCGACAAAGACTGCGAAATGAAGCGCA		attB5_SNAPf
GAAAGCTGGGTTTTAACCCAGCCCAGGCTTG		attB2_SNAPf
GTATACAAAAGTTGTCGTGTCTAAGGGCGAAGAGCTG		attB5_mRuby3
GAAAGCTGGGTTTTACTTGTACAGCTCGTCCATGC		attB2_mRuby3
AAAGCAGGCTTCATGGTGAGCAAGGGCGAGGC		attB1_mScarletI_opt
TGTATACAAAGTTGCCTTGTACAGCTCGTCCATTCCG		attB5r_mScarletI_opt
AGCCGGGCCTTCATCAAGCACCCTGC		mScarletT74I_fw
GAAGGCCCGGCTGCCG		mScarletT74I_rv
AAAGCAGGCTTCATGGCAGACAATTTTTCGCTCCA		attB1_hGal3
TGTATACAAAGTTGCTATCATGGTATATGAAGCACTGGTGAGG		attB5r_hGal3
AAAGCAGGCTTCATGGGGCGCGCGCGC		attB1_hASC
TGTATACAAAGTTGCGCTCCGCTCCAGGTCCTC		attB5r_hASC
CCACCGGAGCTTACCAACTAAACCATGGCCAAGCCT		Blast_pLex_fw
TGTCTCAAGATCTAGAATTCGTTAGCCCTCCCACACATAACCAGAGG		Blast_pLex_rv
CCACCGGAGCTTACCATGAAAAAGCCTGAACTCACCG		Hygro_pLEX_fw
TGTCTCAAGATCTAGAATTCGTCAAGACCAATGCGGAGCATATACG		Hygro_pLEX_rv
CGAATTCTAGATCTTGAGACAAATGGCAGT		pLex307_nopuro_fw
GGTAAGCTCCGGTGGTACCT		pLex307_nopuro_rv
**Recombinant DNA**		
pEGFP-hGal3	Addgene	#73080
pCI-ASC-HA	Addgene	#41553
pKanCMV-mRuby3-10aa-H2B	Addgene	#74258

mNeonGreen-C1	Allele Biotech	
pBAD/His-miRFP670	Addgene	#79984
pSNAPf	New England Biosciences	#E9100S
pEntry-Galectin-3-L1-R5	This work	
pEntry-ASC-L1-R5	This work	
pEntry-ALG-2-L5-L2	This work	
pEntry-LC3B-L5-L2	This work	
pEntry-mNeonGreen-L1-R5	This work	
pEntry-mNeonGreen-L5-L2	This work	
pEntry-mScarlet-L5-L2	This work	
pEntry-mRuby3-L5-L2	This work	
pEntry-SNAPf-L5-L2	This work	
pEntry-mIRFP670-L5-L2	This work	
pLex307-Puro	Addgene	#413922
pLex307-Blast	This work	
pLex307-Hyg	This work	
LentiCrispr v2	Addgene	#52961
pMDL.g/pRRE	Addgene	#12251
pMD2.G	Addgene	#12259
pRSV-Rev	Addgene	#12253
pLex-Hyg-ASC-mNeonGreen	This work	
pLex-Hyg-ASC-mIRFP670	This work	
pLex-Blast-Galectin3-mScarlet	This work	
pLex-Blast-Galectin3-mRuby3	This work	
pLex-Blast-Galectin3-SNAPf	This work	
pLex-Hyg-mNeonGreen-LC3B	This work	
pLex-Hyg-mNeonGreen-ALG-2	This work	
msp12::EBFP2	Addgene	#30177
**Software and Algorithms**		
Graphpad Prism 8.0		graphpad.com/scientific-software/prism/
ImageJ-Fiji – Version: 2.0.0-rc-68/1.52h		imagej.net/Fiji/Downloads
CellProfiler 3.1.5		www.cellprofiler.org
Ilastik 1.3.0		www.ilasik.org
Adobe Illustrator 2019		www.adobe.com
Anaconda Python 3.7		https://www.anaconda.com/distribution/
Custom code for image analysis (Python)	This work	
Avizo	FEI	https://www.fei.com/software/avizo/
Dragonfly 4.0	Object Research Systems	https://www.theobjects.com/dragonfly/
Huygens Professional 18.10		https://svi.nl/Huygens-Professional
TIDE: Tracking of Indels by DEcomposition version 2.0.1		https://tide.deskgen.com/
**Other**		
#1.5 96 well glass bottom plate	Cellvis	Cat #P96-1.5H-N
35mm glass bottom dish	Ibidi	Cat #81218-200
