# Supplementary material for: Plasma membrane damage causes NLRP3 activation and pyroptosis during Mycobacterium tuberculosis infection
Source: Nat Commun. 2020 May 8;11:2270. doi: 10.1038/s41467-020-16143-6 (PMC7210277; doi:10.1038/s41467-020-16143-6)
Supplement: Supplementary file 3 — Description of Additional Supplementary Files [file 41467_2020_16143_MOESM3_ESM.docx]

**Description of Additional Supplementary Files**

**File name:** Supplementary Movie 1

**Description:** Confocal time-lapse imaging of THP1 ASC-GFP (green) infected by Mtb-BFP (blue) undergoing pyroptosis in the presence of DRAQ7 (magenta).

**File name:** Supplementary Movie 2

**Description:** Confocal time-lapse imaging of THP1 ASC-GFP (green) infected by Mtb-BFP (blue) undergoing non-pyroptotic necrosis in the presence of DRAQ7 (magenta).

**File name:** Supplementary Movie 3

**Description:** Confocal time-lapse imaging of THP1 ASC-GFP (green) not infected by Mtb-BFP (blue) undergoing apoptosis in the presence of DRAQ7 (magenta).

**File name:** Supplementary Movie 4

**Description:** Confocal time-lapse imaging of THP1 ASC-GFP (green) infected by Mtb-BFP (blue) undergoing ASC speck formation, pyroptosis and bacterial dissemination, in the presence of DRAQ7 (magenta).

**File name:** Supplementary Movie 5

**Description:** Confocal time-lapse imaging of THP1 ASC-mNeonGreen stained with the mitochondrial membrane potential indicator TMRE (red) and infected by Mtb-BFP (blue).

**File name:** Supplementary Movie 6

**Description:** Confocal time-lapse imaging of THP1 ASC-mNeonGreen (green) GSDMD KD cell, stained with the mitochondrial membrane potential indicator TMRE (red) and infected by Mtb-BFP (blue).

**File name:** Supplementary Movie 7

**Description:** Confocal time-lapse imaging of THP1 ASC-mNeonGreen (green) stained with the mitochondrial membrane potential indicator TMRE (red) and treated with LPS and nigericin.

**File name:** Supplementary Movie 8

**Description:** Confocal time-lapse imaging of THP1 ASC-mNeonGreen (green) GSDMD KD cell, stained with the mitochondrial membrane potential indicator TMRE (red) and treated with LPS and nigericin.

**File name: Supplementary Movie 9**

**Description:** Confocal time-lapse imaging of THP1 ASC-GFP (green)/Gal3-mRuby3 (magenta) infected by Mtb-BFP (blue).

**File name: Supplementary Movie 10**

**Description:** Confocal time-lapse imaging of THP1 mNeonGreen-LC3B (green)/Gal3-mScarlet (magenta) infected by Mtb-BFP (blue).

**File name: Supplementary Movie 11**

**Description:** Confocal time-lapse imaging of THP1 Gal3-mScarlet (magenta) stained by LysoView633 (green) and infected by Mtb-BFP (blue).

**File name: Supplementary Movie 12**

**Descipriton:** Confocal time-lapse imaging of THP1 Gal3-mScarlet (magenta) stained by LysoView633 (green) and infected by Mtb-BFP (blue).

**File name: Supplementary Movie 13**

**Description:** Confocal time-lapse imaging of THP1 ASC-GFP (red) stained by LysoView633 (green) and infected by Mtb-BFP (blue).

**File name: Supplementary Movie 14**

**Description:** Confocal time-lapse imaging of THP1 mNeonGreen-ALG-2 (green)/Gal3-mScarlet (magenta) infected by Mtb-BFP (blue).

**File name: Supplementary Movie 15**

**Description:** Confocal time-lapse imaging of THP1 mNeonGreen-ALG-2 (green)/Gal3-mScarlet (magenta) infected by Mtb-BFP (blue).

**File name: Supplementary Movie 16**

**Description:** Simultaneous widefield and TIRF time-lapse imaging of THP1 mNeonGreen-ALG-2 (green)/Gal3-mScarlet (magenta) infected by Mtb-BFP (blue).

**File name: Supplementary Movie 17**

**Description:** Widefield time-lapse imaging of THP1 mNeonGreen-ALG-2 (green) stained with Calbryte-590 calcium indicator (grey) and infected by Mtb-BFP (blue).

**File name: Supplementary Movie 18**

**Description:** Widefield time-lapse imaging of THP1 Gal3-SNAP(SiR) (magenta) stained with Calbryte- 590 calcium indicator (grey) and infected by Mtb-BFP (blue).

**File name: Supplementary Movie 19**

**Description:** Confocal time-lapse imaging of THP1 mNeonGreen-ALG-2 (green) in the presence of extracellular propidium iodide (magenta) and infected by Mtb-BFP (blue).

**File name: Supplementary Movie 20**

**Description:** Confocal time-lapse imaging of THP1 mNeonGreen-ALG-2 (green)/Gal3-mScarlet (magenta)/ASC-mIRFP670 (grey) infected by Mtb-BFP (blue).

**File name: Supplementary Movie 21**

**Description:** Simultaneous widefield and TIRF time-lapse imaging of THP1 mNeonGreen-ALG-2 (green)/Gal3-mScarlet (magenta) stimulated by silica.

**File name: Supplementary Movie 22**

**Description:** Confocal time-lapse imaging of THP1 mNeonGreen-ALG-2 (green)/Gal3-mScarlet (magenta)/ASC-mIRFP670 (grey) stimulated by silica.

**File name: Supplementary Movie 23**

**Description:** Confocal time-lapse imaging of THP1 mNeonGreen-ALG-2 (green)/Gal3-mScarlet (magenta)/ASC-mIRFP670 (grey) stimulated by LPS and nigericin.

**File name: Supplementary Movie 24**

**Description:** Confocal time-lapse imaging of THP1 mNeonGreen-ALG-2 (green)/Gal3-mScarlet (magenta)/ASC-mIRFP670 (grey) stimulated by LPS and imiquimod.

**Name: Supplementary Movie 25
Description:** Simultaneous widefield and TIRF time-lapse imaging of THP1 mNeonGreen-ALG-2 (green, TIRF and WF)/Gal3-mScarlet (magenta, TIRF and WF)/ASC-mIRFP670 (grey, WF), loaded with TPSC2a and exposed to 405 nm blue light prior to imaging.
